# Cervical intervertebral disc disease in 60 Yorkshire terriers

**DOI:** 10.3389/fvets.2023.1148802

**Published:** 2023-05-12

**Authors:** Viktor Palus, Ladislav Stehlik, Alois Necas, Robert Srnec, Lucie Urbanova, Diane Lu

**Affiliations:** ^1^Neurovet, Trencin, Slovakia; ^2^Faculty of Veterinary Medicine, University of Veterinary Sciences Brno, Brno, Czechia; ^3^CityU Veterinary Medical Centre, Kowloon, Hong Kong SAR, China

**Keywords:** cervical intervertebral disc extrusion, canine, ventral slot, outcome, prevalence

## Abstract

Intervertebral disc extrusion (IVDE) is a common neurological condition in many dog breeds. This study aimed to describe this condition in Yorkshire terriers (YT) and calculate the prevalence of this condition amongst the YTs with neurological diseases. This is a double-centre retrospective study which was conducted in two arms. The first part of the study, describing the clinical features and prognosis of cervical (C) IVDE in YTs, is based on data from 2005 to 2021. The second part of the study calculated the prevalence of C IVDE amongst the YTs with neurological diseases based on data from 2016 to 2021. A retrospective search through the medical records was conducted. YTs with C IVDE diagnosed with MRI and confirmed surgically were eligible for inclusion in this study. Sixty YTs were included in the first part of the study. There were 48 (80%) dogs with acute onset and 12 (20%) with chronic onset with acute deterioration. Ambulation was preserved in 31 (51.7%) dogs on admission, and the remaining 29 (48.3%) dogs were non-ambulatory. No significant association was found between ambulation on admission and recovery status (*p* = 0.547). Seventy-three intervertebral spaces were treated during the surgical intervention. Relapses were seen in seven (11.7%) dogs. Forty-nine (81.7%) dogs were ambulatory at discharge. A complete recovery was observed in 46 (76.7%) dogs; the remaining dogs (14, 23.3%) were classified as incomplete recovery. A significant difference was found in time to ambulation (*p* = 0.0238) and time to discharge (*p* = 0.0139) between the on-admission ambulatory and non-ambulatory dogs. Three hundred and eight YTs were diagnosed with neurological diseases between 2016 and 2021 in one referral centre. C IVDE was diagnosed in 31 (10.06%) dogs. This is the first study explicitly describing the C IVDE in YTs and establishing the prevalence of this condition amongst YTs with other neurological disorders.

## Introduction

Yorkshire terriers (YT) are the 13th most popular breed according to the ranking of the American Kennel Club for 2021 (1). To our knowledge, a similar ranking has yet to be done in Slovakia, the Czech Republic, or in Hong Kong; however in our experience, this breed is quite popular in these countries.

YTs are predisposed to various neurological diseases, but despite their popularity, no specific studies regarding neurological conditions have been done on this breed. According to one study (2), neurological diseases were the third most common cause of death in YT. Unfortunately, specific neurological diseases responsible for deaths were not listed (2). The most described neurological diseases throughout the veterinary literature in YTs are necrotising meningoencephalitis (3), necrotising leucoencephalitis (4), atlantoaxial subluxation and instability (5), congenital hydrocephalus (6), mitochondrial encephalopathy (7) or L-2-hydroxyglutaric aciduria (8). Even though YT is listed as a chondrodystrophic breed (9), there are only a few cases of intervertebral disc disease in YTs reported in veterinary literature (10–13).

Intervertebral disc disease is a common neurological problem in dogs (14). The estimated lifetime prevalence of intervertebral disc diseases in the general dog population is 2–3.5% (15, 16). Dachshunds are generally over-represented in studies, with the majority of cases affected by thoracolumbar (TL) intervertebral disc extrusions (IVDE) (9, 16, 17). A detailed description of the clinical course, treatment and prognosis of this condition has also been described in different breeds of dogs (18*–*20). Approximately 15% of cases with intervertebral disc herniation are localised in the cervical region (21, 22). Some breeds, like Beagles and Shih Tzus, are more commonly affected by cervical intervertebral disc herniations (11). We observe that YTs are more commonly affected with C IVDE than can be expected from the literature.

Hence, the aim of this study was to describe the clinical features and prognosis of YTs with this condition. Further, we aimed to determine the prevalence of C IVDE in the population of YTs with neurological diseases. Finally, we hypothesised that YTs suffer from C IVDE and have a good prognosis with surgical treatment.

## Materials and methods

This is a double-centre retrospective clinical study which was conducted in two arms. The study was approved by Animal Experimentation Ethics Committee at the University of Veterinary Sciences Brno (*REFNO: ES_11-2022_Necas*). The first part of the study, describing the characteristics of C IVDE in YTs, is based on data between 2005 and 2021. This part of the study involved cases from two referral practices in Slovakia and Hong Kong. The second part of the study that calculated the prevalence of C IVDE amongst the YTs with neurological diseases is based on data from 2016 to 2021. In this part of the study, all YTs with neurological conditions presented to one referral centre in Slovakia were used for calculation.

Only YTs with C IVDE diagnosed with MRI and confirmed surgically were eligible for inclusion in this study. A retrospective search through the medical records was conducted. We analysed a signalment, onset and progression of the presented neurological symptoms, neurological examination, magnetic resonance imaging (MRI) findings, lesion localisation, surgical site correction, a complication of the surgery, time to ambulation after surgery, time to discharge after surgery, outcome defined as complete or incomplete recovery, relapses and death if occurring before the time of finalising the data of the study. The words used for searching throughout the records were: “Yorkshire Terrier,” “intervertebral disc extrusion,” and “ventral slot.” We excluded cases in which the medical records were incomplete; follow-up was not carried out within 1 month after the surgery and cases in which the extruded intervertebral disc material was not identified in the vertebral canal. The onset of the neurological signs was characterised as acute when owners started noticing and reporting the first clinical signs with a duration less than 10 days before the first visit at the referring veterinary surgeon. Clinical signs lasting longer than 10 days with episodic progression were characterised as chronic onset (23). For this study, we formed a third group: “chronic onset with acute deterioration.” This category contains all the cases with the chronic onset of clinical signs with acute deterioration of the clinical signs. Days from the first day of the tentative onset of the clinical signs to the neurological examination by the specialist in veterinary neurology (VP, DL) were noted. Neurological symptoms were graded on a scale from 1 to 3. Grade 1 was defined as neck pain with no neurological deficits; grade 2 was defined as ambulatory tetraparesis with or without signs of neck pain; grade 3 was defined as non-ambulatory tetraparesis or tetraplegia with or without signs of neck pain (24). Neck pain was identified when a patient showed cervical hyperaesthesia during the neurological examination. A board-certified neurologist (VP, DL) performed a neurological exam and surgery. Only dogs with ventral slots performed were included in the study. The intervertebral disc material compressing the spinal cord had to be identified and removed during the surgery. Follow-up consultation with a board-certified neurologist (VP, DL) was conducted at least 7 days to 1 month after the surgery. Magnetic resonance units used in this study were 0.18 T (VET-MR, ESAOTE, Genova, Italy), 0.2 T (General Electric MR Goldseal, United States) and in 6 cases 1.5 T (General Electric MR Signa Explorer, United States). Minimal T2 weighted sagittal and transverse images were used to diagnose the affected site as previously recommended for diagnosing TL IVDE (25). A complication of the surgery was defined as any perioperative problem encountered during the surgery or lack of improvement after the surgery whether, requiring re-operation or not. Ambulation was defined as the ability of the patient to rise and to be able to perform ten unassisted, consecutive, weight-bearing steps (26). The attending board-certified neurologist (VP, DL) evaluated the completeness of the recovery at the follow-up consultation. Complete recovery was identified as having ambulatory status with no detected neurological deficits. Incomplete recovery was identified as having ambulatory status with detected neurological deficits (general proprioceptive ataxia, ambulatory tetraparesis). Relapses were defined as recurrence of cervical hyperaesthesia or neurological signs that occurred longer than 1 month after the surgery. Not all relapses were further diagnosed or surgically treated, but the presence of the symptoms mentioned above was already counted as relapse. Death, due to a related or unrelated cause, was recorded if it happened before completing data collection.

### Statistical analysis

Statistical analysis was performed in R, a free software environment for statistical computing and graphics using Rstudio (27, 28). Data distribution was not an issue due to the sample size and was not tested. The associations between categorical data were tested by the chi-squared or Fisher’s exact test. The quantitative variables were compared with a two-sample t-test. The level of statistical significance was set at 0.05.

## Results

Sixty YTs were included in the first part of the study. There were four (6.7%) intact females, nine (15.0%) neutered females, 33 (55.0%) intact males and 14 (23.3%) neutered males. No significant association was found between sex and any categorical variable (*p* > 0.05). The mean ± SD weight at the time of surgery was 5.0 ± 1.76 kg. The mean ± SD age at the time of surgery was 112.2 ± 35.34 months (9.3 ± 2.94 years).

All but five dogs had neurological deficits on the initial examination (91.6%). Five dogs (8.3%) were presented only with cervical hyperaesthesia with no neurological deficits and were included in Grade 1. Grade 2 included 26 (43.3%) dogs, and Grade 3 included 29 (48.4%) dogs. Fifty-four (90%) of the dogs had neck pain on clinical examination, and the remaining six (10%) dogs showed no signs of neck pain. Ambulation was preserved in 31 (51.7%) dogs on admission, and the remaining 29 (48.3%) dogs were non-ambulatory. No significant association was found between ambulation on admission and recovery status (*p* = 0.547).

There were 48 (80%) dogs with acute onset and 12 (20%) with chronic onset with acute deterioration. None of the dogs were eligible for inclusion into the chronic group. The descriptive statistics for the deterioration duration for both groups are displayed in Table 1. A significant difference was observed (*p* = 0.0195) in the deterioration duration between the two types of deterioration. It means that the duration of presenting symptoms in the actue group was significantly shorter than in the group of chronic onset with acute deterioration. No significant association was observed (*p* = 0.337) between ambulation status and deterioration type. However, a significant difference (*p* = 0.00497) was found in deterioration duration between the ambulatory and non-ambulatory groups. The mean (±SD) deterioration duration in the ambulatory and non-ambulatory groups was 36.1 (±53.50) days and 6.4 (±11.99) days, respectively.

**Table 1:** Descriptive statistics of days from the onset of the clinical signs to the presentation at the referral centre (*n* = 60).

Deterioration type	*n*	Mean	SD	Median	Minimum	Maximum
Acute	48	9.9	13.59	5.5	1	60
Chronic onset with acute deterioration	12	68.8	74.54	30.0	1	180

Seventy-three intervertebral spaces were treated during the surgical intervention. Forty-nine (81.67%) dogs had one intervertebral space, nine (15.0%) dogs had two intervertebral spaces, and two (3.33%) dogs had three intervertebral spaces treated surgically. The average number of surgically treated C IVDE was 1.2 spaces per dog. The most common surgically treated intervertebral space was C4-C5. The frequency of individual intervertebral space is displayed in Table 2.

**Table 2:** Frequency of intervertebral spaces treated surgically (*n* = 73).

Location	C2-3	C3-4	C4-5	C5-6	C6-7	C7-T1
*n*	3	23	28	9	7	3
Percent	4.11	31.51	38.36	12.33	9.59	4.11

**Figure 1:**
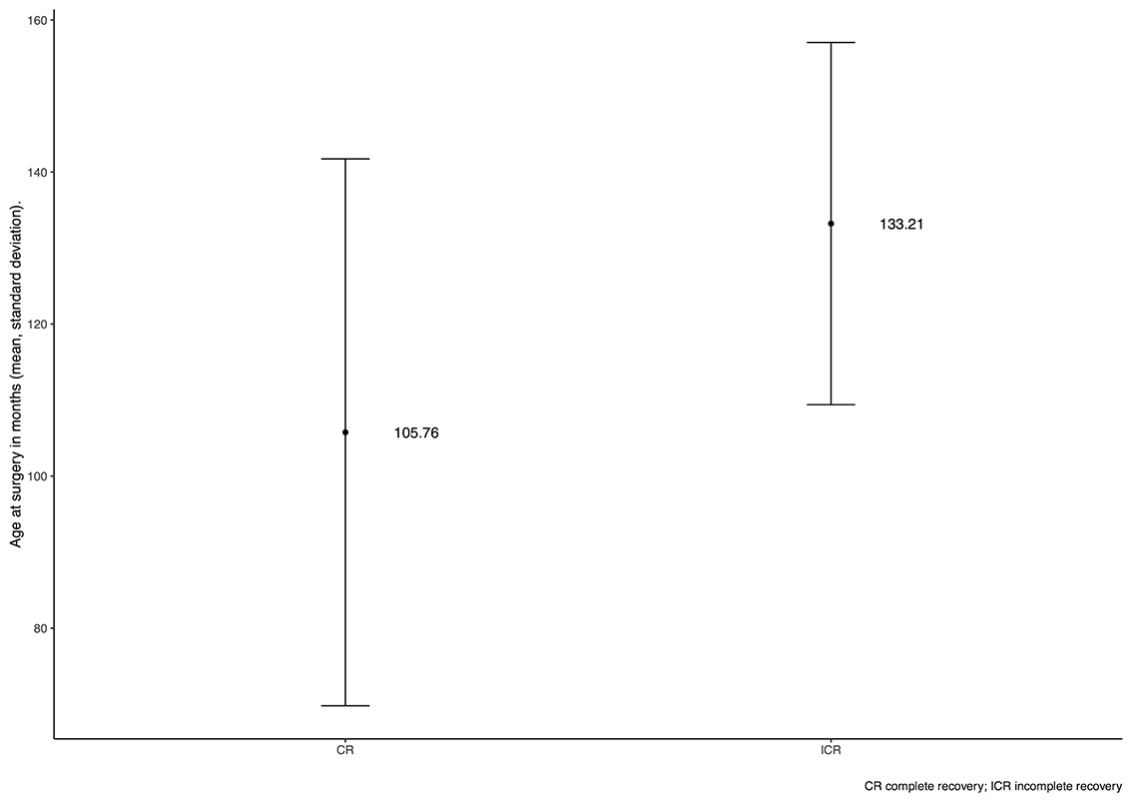
Bar plot of the patient’s age at the time of surgery for a group of dogs with complete and incomplete recovery. The central point represents the mean, and the lines represent SD. CR, complete recovery; ICR, incomplete recovery.

All dogs regained ambulation at some point after the surgery. Forty-nine (81.7%) dogs were ambulatory at discharge. Four dogs that were ambulatory prior to surgery became temporarily non-ambulatory after the surgery. We consider these case as a complication of the surgery. Hence, the surgical complication rate accounts for 6.7%. Despite this temporary complication all ambulatory dogs at the admission were also ambulatory at the time of discharge. Of 29 non-ambulatory dogs at the admission 18 were ambulatory at the time of discharge. A complete recovery was observed in 46 (76.7%) dogs. Fourteen dogs (23.3%) were classified as incomplete recovery but still were ambulatory. The age of dogs in these two groups was significantly different (Figure 1). Relapses were seen in seven (11.7%) dogs.

**Figure 2:**
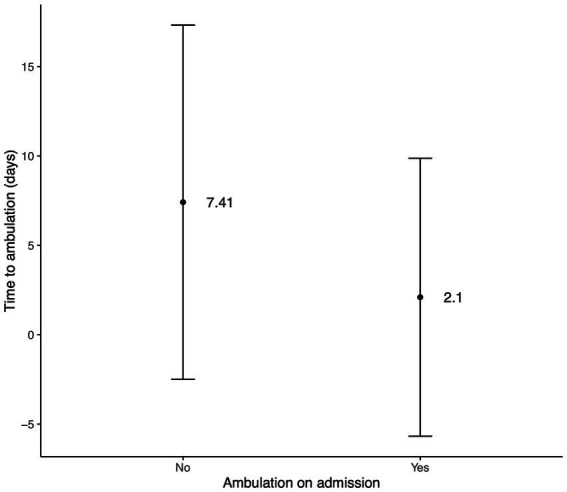
Bar plot of days to ambulation after surgery regarding the ambulation on admission. The central point represents the mean, and the lines represent SD.

**Figure 3:**
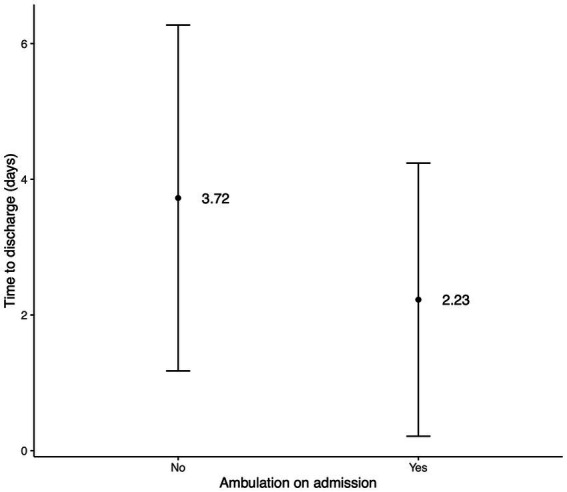
Bar plot of days to discharge after surgery regarding the ambulation on admission. The central point represents the mean, and the lines represent SD.

Descriptive statistics for time to ambulation and discharge after surgery are recorded in Tables 3, 4. A significant difference was found in time to ambulation (*p* = 0.0238, Figure 2) and time to discharge (*p* = 0.0139, Figure 3) between the on-admission ambulatory and non-ambulatory dogs.

**Table 3:** Descriptive statistics for the time to ambulation after the surgery for the entire study group and ambulatory and non-ambulatory groups.

Categorical variable	What was measured	n	Mean	SD	Median	Minimum	Maximum
Time to ambulation	Days to ambulation after the surgery	60	4.7	9.19	1	0	50
Time to ambulation: non-ambulatory (grade 3) on admission		29	7.4	9.91	4	0	50
Time to ambulation: ambulatory (grades 1 and 2) on admission		31	2.1	7.77	0	0	43

**Table 4:** Descriptive statistics for the time to discharge after the surgery for the entire study group and ambulatory and non-ambulatory groups.

Categorical variable	What was measured	n	Mean	SD	Median	Minimum	Maximum
Time to discharge	Days to discharge after the surgery	60	2.9	2.39	2	1	13
Time to discharge: non-ambulatory (grade 3) on admission		29	3.7	2.55	3	1	13
Time to discharge: ambulatory (grades 1 and 2) on admission		31	2.2	2.01	1	1	9

Three hundred and eight YTs were diagnosed with neurological diseases between the years 2016 and 2021 in the referral centre in Slovakia. Out of these patients, we collected 45 (14.6%) surgically confirmed IVDE in all locations of the vertebral column. C IVDE was diagnosed in 31 (10.06%) out of all 308 dogs. Two hundred and seventy-seven (89.94%) dogs had neurological diagnoses other than surgically confirmed C IVDE. The age and weight of dogs in these two groups were significantly different. Dogs with C IVDE were older and heavier. No significant difference in sex was observed between these groups.

## Discussion

This study retrospectively describes the clinical characteristics of YTs with C IVDE. The results confirm our hypothesis that C IVDE occurs often among the YTs with neurological problems. Some studies report that C IVDEs account for around 15% of all IVDE cases in dogs (21, 22). The prevalence of C IVDE in our study within the group of YTs with other neurological conditions was 10.06%. The prevalence is relatively high, although it must be noted that it is calculated only from the YTs that presenting with neurological problems. Therefore, the true prevalence within the general population of YTs might be lower. Mildly higher prevalence of C IVDE was also reported in French Bulldogs with a neurological condition in which the intervertebral disc herniation localised in the cervical area accounted for 18.6% (29). Different publications claim many different breeds to be predisposed to C IVDE. However, true prevalence of C IVDE for these breeds cannot be reliably drawn from these publications (11, 17, 30–33). YTs were reported to account for 6.1% of all dogs with C IVDE and only 0.6% of all dogs with TL IVDE (10). It is worth mentioning that amongst 308 YTs with neurological diseases, only 14 YTs were diagnosed and surgically confirmed with thoracolumbar IVDE. We also identified 22 other YTs with C intervertebral disc disease and treated conservatively from the population of 308 YTs. However, we did not include them in our study as one of the inclusion criteria was surgical confirmation of the extruded material into the vertebral canal. The reason for this was the inability to validate that these cases were genuine IVDEs rather than different types of intervertebral disc disorders (34).

The definition of chondrodystrophy varies throughout the literature. YT has been considered a non-chondrodystrophic breed in one publication (11). In another publication describing the expression of FGF4 in domestic dogs, breeds were considered chondrodysplastic if they met all their set criteria. This publication identified YTs as carrying the insertion containing FGF4 retrogene at a higher frequency than expected since they did not show the characteristic chondrodysplastic phenotype. This observation was made only on 4 YTs, but half of them carried the insertion mentioned above (35). Two separate FGF4 retrogenes were described on canine chromosomes CFA 12 and CFA 18 (36). Some of the breeds most susceptible to IVDE carry retrogenes on both CFA 12 and CFA 18. The expression of a retrogene on CFA 12 was the major contributor associated with intervertebral disc surgery (37). It must be noted that these breeds often also carry the retrogene on CFA 18 (36–38). However, this retrogene on CFA 18 is missing in other predisposed breeds, such as Beagles, French Bulldogs and Cocker Spaniels (37). Phenotypically similar dogs to the predisposed breeds, like Cairn Terriers or West Highland White Terriers, carry the retrogene only on CFA 18 and rarely present with IVDE (36–38).

YTs have been reported to carry retrogenes on both CFA 12 and CFA 18. However, the authors are aware of only about 12 animals in the literature, out of which only one animal carried one copy of CFA 12 FGF4 retrogene. Other YTs either carried no copies on CFA 12 or two FGF4 retrogene copies on CFA 18 (37).

We cannot sufficiently explain why YTs are predisposed to C IVDE even though they do not have typical overexpression of retrogene FGF4 on CFA 12 as it is reported in other predisposed breeds. Our study shows that YTs are predisposed mainly to C IVDE. Batcher et al. showed that FGF4 retrogene on CFA 12 was the main contributor associated with IVD surgery. In that study, 25.1% of the surgeries were performed in the area between C1 and T1. That study also included only 12 YTs who underwent surgical treatment for IVDE. This study does not further describe whether these YTs were surgically treated in the cervical area or other vertebral column areas (37). One can only hypothesise that some of these YTs were surgically treated in the cervical area similarly to our study population, and hence they only rarely expressed FGF4 retrogene on CFA 12. We did not investigate the expression of FGF4 in our study population. It is one of the main limitations of this study which is unavoidable mainly due to the retrospective nature of this study. The study began before the causative role of FGF4 retrogene was identified in the veterinary literature. Moreover, this was a clinical study, and genetic investigation would not change the treatment strategy and outcome of the affected patients. It could not be clinically or financially justified to the owners.

Our study identified that males were more commonly affected with C IVDE and presented 78.3% of all cases. This contrasts with one study containing 12 YTs where males and females were equally presented (11). In addition, two studies looking at the cohort of different breeds identified that males were more commonly affected with C IVDE than females (10, 13). Still, other studies refute this with sex-equal distribution (11, 30–33). One study also concluded that males and neutered dogs had a higher prevalence of C IVDE causing non-ambulatory status (13), but we did not see any association between the sex or neutering status and any categorical variable (*p* > 0.05).

The mean ± SD weight at the time of surgery in our study was 5.0 ± 1.76 kg, more than the 3.4-kg reported in two studies (10, 11). The mean ± SD age at the time of surgery was 9.3 ± 2.94 years in our study which is similar to YTs in previous publications (10, 11). The age at the time of the surgery in YTs appears to be higher than the previously reported age of small breeds with 8.2 years, 8.1 years, and 7.8 years from previous studies (10, 11, 31). Only one YT was younger than 4 years in our population. It is, therefore, unlikely that YTs younger than 4 years would be clinically affected by C IVDE. This is in accordance with a study that suggests that YTs may be prone to developing C IVDE at an older age (11).

YTs in our publication showed neurological deficits with signs falling into grade 2 and grade 3 in 43.3 and 48.4%, respectively. Only 8.3% of our population presented with signs falling into grade 1 (neck pain). This contrasts with other studies in which cervical hyperaesthesia with no neurological deficits was more commonly encountered (10, 11, 31, 32). Cervical hyperaesthesia was the only clinical sign in 33–58% (10, 11, 31) of surgically treated and in 76% of conservatively treated dogs with C IVDE (32). Fifty-four (90%) of YTs throughout all the grades in our study had neck pain on the neurological examination. Cervical hyperaesthesia was also the most common clinical sign found in 87% of all dogs with or without neurological deficits (31). This is unsurprising because cervical hyperaesthesia is one of the leading findings in dogs with C IVDE (23, 34, 39). Cervical hyperaesthesia, on the other hand, is not that common in different disc-related diseases, such as intervertebral disc protrusion (34, 39) or hydrated nucleus pulposus extrusion (34, 40). Ambulation (grades 1 and 2) was preserved in 31 (51.7%) dogs on admission, and the remaining 29 (48.3%) dogs were non-ambulatory (grade 3). Non-ambulatory status in our study is higher than 16, and 25% of YTs reported to be non-ambulatory in two studies (10, 11). Different studies report various numbers of non-ambulatory dogs on admission, ranging from 3% in the publication focusing on conservatively treated dogs (32) to 100% in the publication focusing on surgically treated dogs (41). However, publications with a larger cohort of dogs reported between 25 and 45% of dogs as non-ambulatory on admission (11, 13, 31). This discordance is likely due to selection bias in the studies. Our study may also have a similar bias because we included only cases referred to neurological referral centres. Many YTs with C IVDE and milder neurological deficits were not referred to us and hence were not considered in our population. No significant association was found in our study for ambulation on admission and recovery status (*p* = 0.547). However, a significant difference was found in time to ambulation (*p* = 0.0238) and time to discharge (*p* = 0.0139) between the on-admission ambulatory and non-ambulatory dogs. According to these results ambulatory dogs on admission started walking and were discharged earlier than the dogs that were non-ambulatory on the admission.

Most YTs in this study were presented with acute onset of the clinical signs (80%), and the rest of were presented with a history of chronic onset with acute deterioration (20%). The mean duration of clinical signs in a group of dogs with acute onset was 9.9 days. A significantly (*p* = 0.0195) longer period of 68.8 days was recorded for dogs with chronic onset with acute deterioration. This differs from one study where acute deterioration was not so common and occurred in 45%. However, the acuteness and chronicity of the clinical signs in that study were defined as the clinical signs developing over a period of <24 h and >24 h, respectively (11). This is quite stringent definition and we used 10 days cut-off between acute and chronic onset of clinical signs (23). It was evident that despite the chronic onset of the neurological signs in 20% of YTs in this study, all of them eventually had acute deterioration rendering them to be referred and surgically treated. Therefore, we formed a third group of “chronic onset with acute deterioration” to better show the temporal development of the clinical signs. No significant association was observed (*p* = 0.337) between ambulation status and deterioration type. However, a significant difference (*p* = 0.00497) was found in deterioration duration between the ambulatory and non-ambulatory groups. The mean (±SD) deterioration duration in the ambulatory and non-ambulatory groups was 36.1 (±53.50) days and 6.4 (±11.99) days, respectively.

Seventy-three intervertebral spaces in 60 YTs were treated during the surgical intervention. YTs had, on average, two affected discs per dog in two large cohorts of cases (10, 11). It was also suggested that YTs are commonly affected with C IVDE in multiple sites compared to other breeds. However, another study refuted this and reported YTs to be commonly affected by a single IVDE (13). Our study showed that most YTs had a single site of IVDE, but multiple locations are not uncommon in YTs either.

The most affected disc space in our study was C4-C5, followed by C3-C4 and C5-C6, respectively. However, larger cohort studies reported C5-C6 disc space as the most frequently affected in the general breed population (13, 41). This finding is comparable to our results and suggests more caudal distribution of affected disc spaces in YTs.

Our study identified seven YTs with reported relapses, accounting for 11.7% of all cases. This is in line with the previously reported rate of relapses for surgically treated dogs with C IVDE, which was 10–16% (31, 41, 42). However, other studies reported a higher rate of relapses accounting for 33–40%. This discrepancy compared to our study might be caused by the fact that most of these dogs, although not all, were treated conservatively (32, 43). Another reason for this can be the lack of follow-up diagnostic work-up for dogs in our study.

All YTs in this study regained ambulatory status (100%) at some point after the surgery. Forty-nine (81.7%) dogs were ambulatory at discharge. This is in line with other studies that report the recovery of ambulation between 96.2 and 100% after surgery (13, 31). We defined the recovery status into two groups, complete or incomplete. Incomplete is the ambulatory status with detected residual neurological deficits on neurological examination done by a board-certified neurologist (VP, DL). A complete recovery was observed in 46 (76.7%) dogs; the remaining 14 dogs (23.3%) were classified as incomplete recovery. This aligns with other studies in which complete recovery was achieved in 59.5 and 62% of the surgically treated dogs (13, 41). The age between the dogs achieving complete and incomplete recovery differed significantly; the younger ones were more likely to achieve complete recovery.

The mean time to ambulation after the surgery in our population of YTs was 4.7 days (range 0 to 50 days). The YTs who were ambulatory on admission had a significantly shorter time to ambulation (mean 2.1 days) compared to non-ambulatory ones (mean 7.4 days). Four dogs in our study deteriorated after the surgery. Although this deterioration was temporary. Hence, we consider this as a minor complication of the surgery which occurred in 6.7% of the cases. These findings are in line with previously reported ambulation times after the surgery. The median range for dogs reaching the ambulation was between 3 to 14 days (11, 13, 31, 41). Dogs with lesser neurological deficits were also expected to have a shorter time to ambulate. This is logical and aligns with other studies (31, 32).

The mean and median time to discharge of YTs after the surgery in our study was 2.9 and 2 days (range 1–13), respectively. Dogs that were ambulatory on admission had a significantly shorter time of hospitalisation.

The main limitation of the study was its retrospective nature. It would be interesting to know the FGF4 retrogene status of the YTs in our study. Another limitation is that the group of YTs consists of cases from two institutions in which two different neurologists could have had a potentially different opinion on neurological deficits. Hence some of the results could be individually biased, mainly regarding the inclusion criteria, the decision regarding the discharge time and the achievement of complete vs. incomplete recovery. The lack of follow-up imaging in relapsing cases is another limitation of this study. Repeating MRI in these cases could better identify reasons of relapses with potentially different therapeutic strategy of these cases. The limitation of the study is also the need for the inclusion of the entire YT population in the calculation of the prevalence. It would be more precise to examine at least the YTs with any medical condition and to calculate the prevalence. This is again caused by the retrospective nature of this study and the lack of access to this information.

We conclude that C IVDE occurs often in YT. According to this study one in ten YTs with a neurological condition was surgically treated with C IVDE. The prognosis for surgically treated YTs with C IVDE is good and complete recovery can be achieved in most cases. Younger dogs have a better prognosis when assessing the completeness of the recovery compared to older ones. It is unlikely that YTs younger than 4 years would be presented with C IVDE. We can also conclude that ambulatory dogs before surgery have a shorter hospitalisation time. No other variables were significantly associated with better or worse outcomes or hospitalisation time.

## Data availability statement

The raw data supporting the conclusions of this article will be made available by the authors, without undue reservation.

## Ethics statement

The animal study was reviewed and approved by Animal Experimentation Ethics Committee, VETUNI. Written informed consent was obtained from the owners for the participation of their animals in this study.

## Author contributions

VP and DL conceived and designed the present study and collected the data. VP, LS, and AN analysed the data and wrote the draft manuscript. VP, LS, AN, RS, LU, and DL performed the critical revisions of the manuscript. All authors contributed to the article and approved the submitted version.

## Conflict of interest

The authors declare that the research was conducted in the absence of any commercial or financial relationships that could be construed as a potential conflict of interest.

## Publisher’s note

All claims expressed in this article are solely those of the authors and do not necessarily represent those of their affiliated organizations, or those of the publisher, the editors and the reviewers. Any product that may be evaluated in this article, or claim that may be made by its manufacturer, is not guaranteed or endorsed by the publisher.
